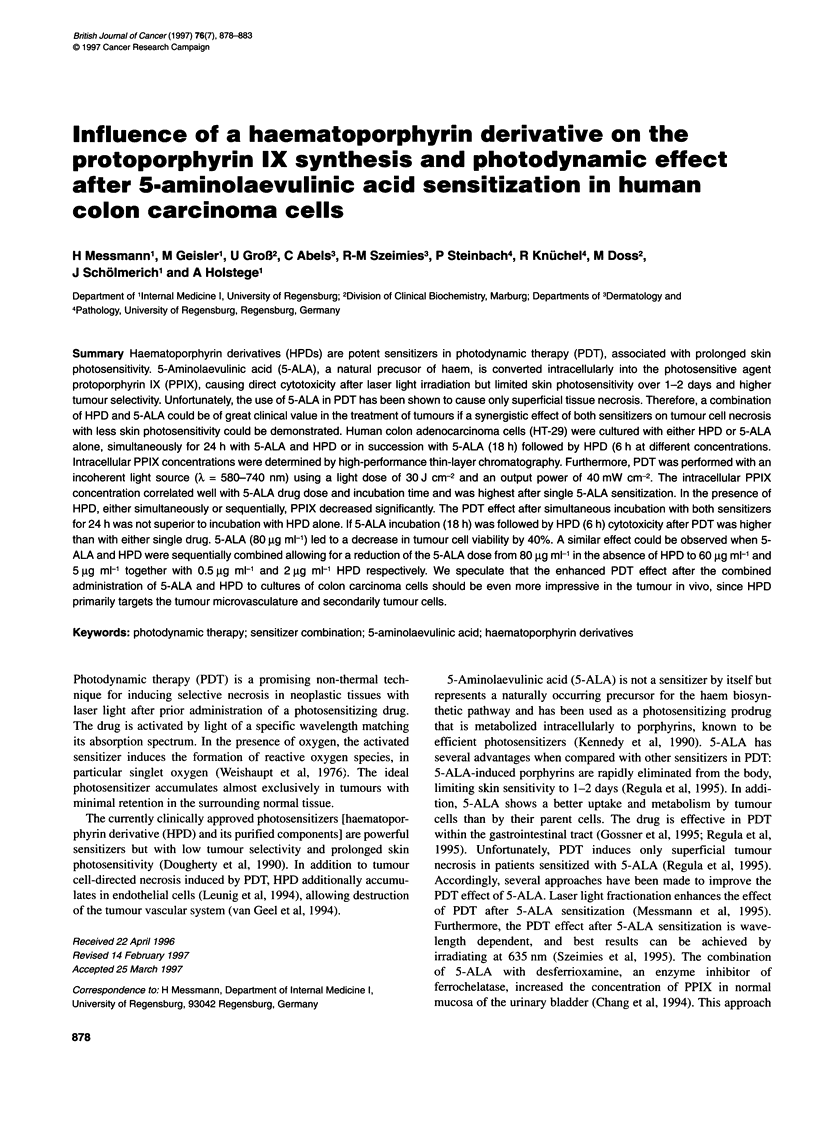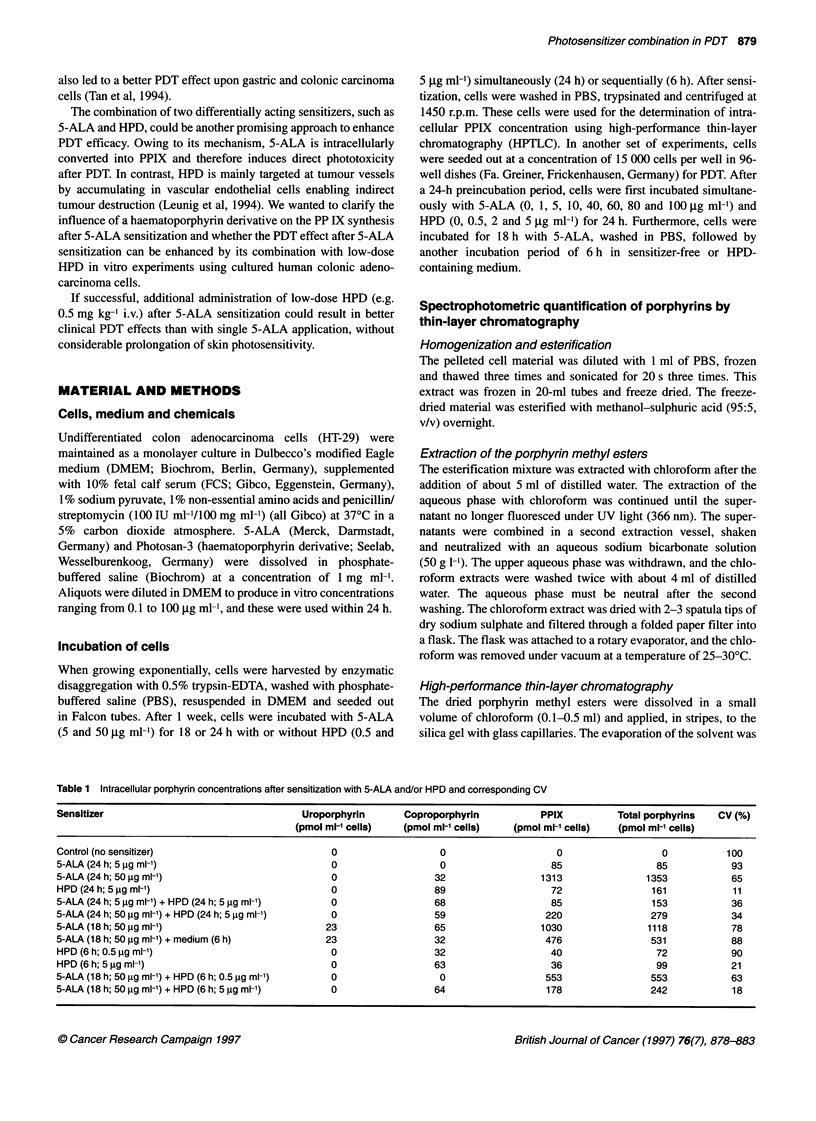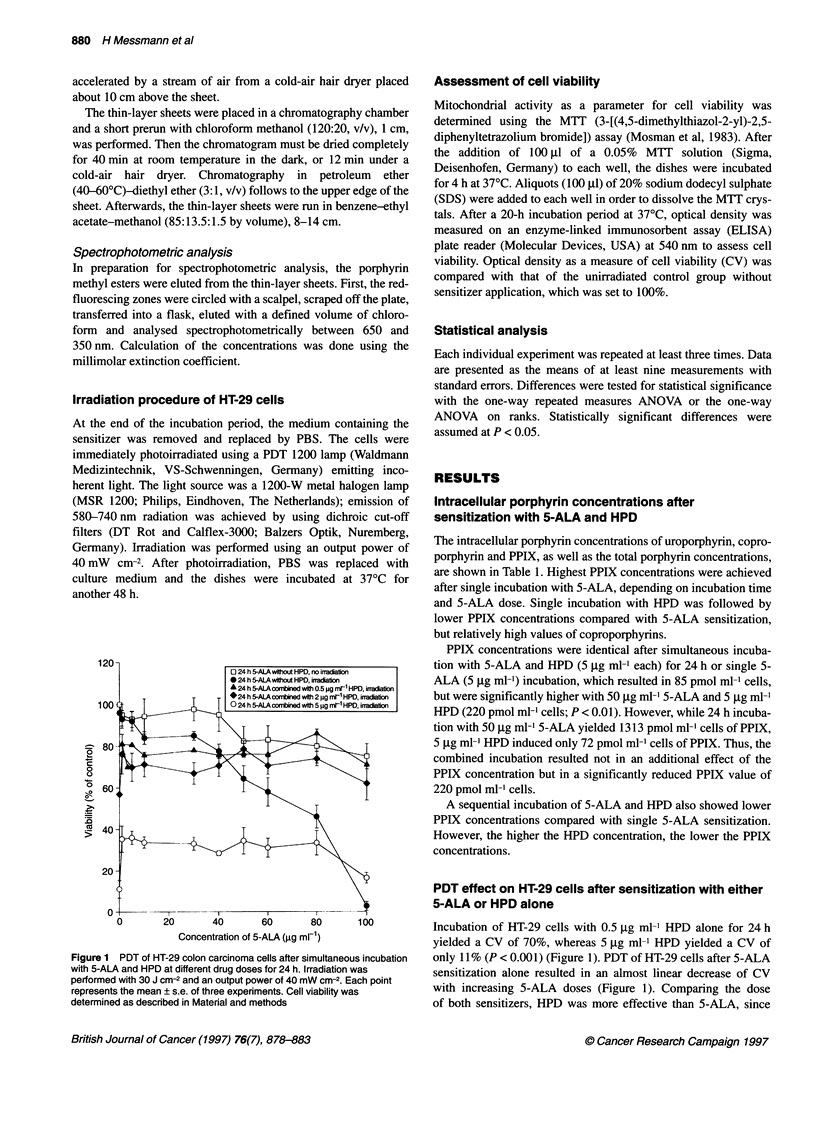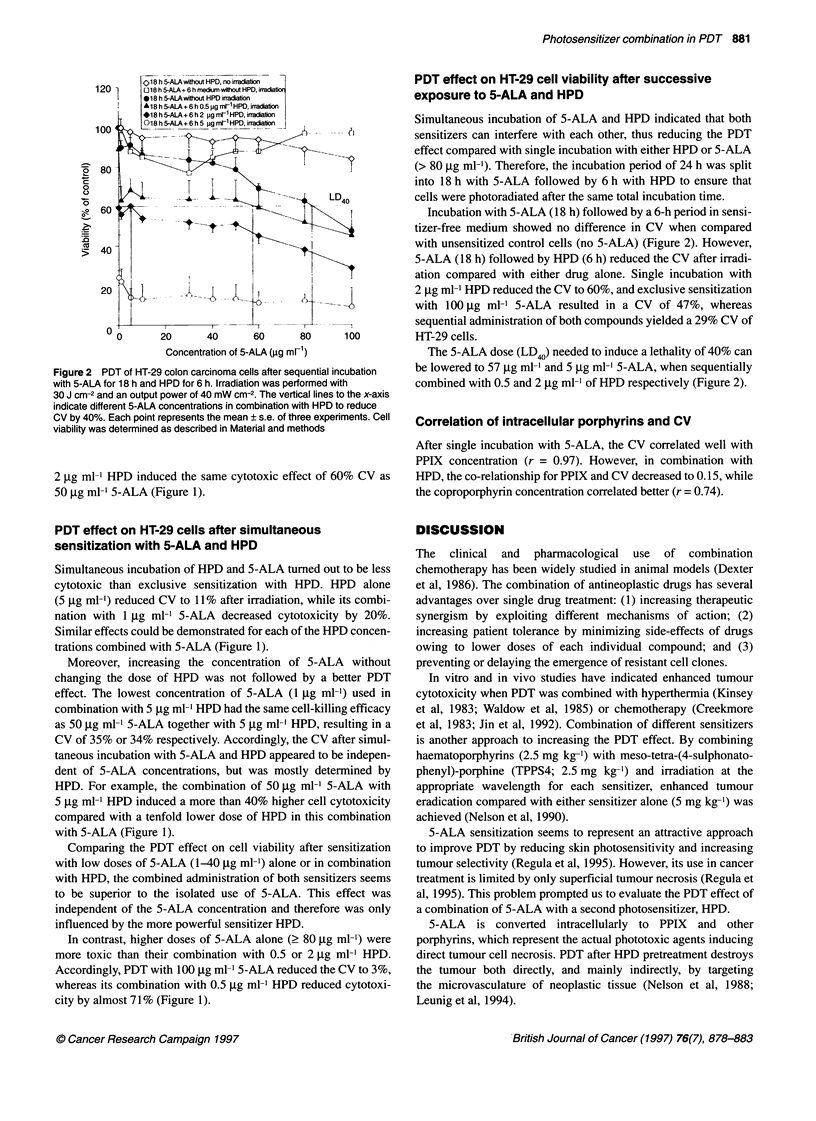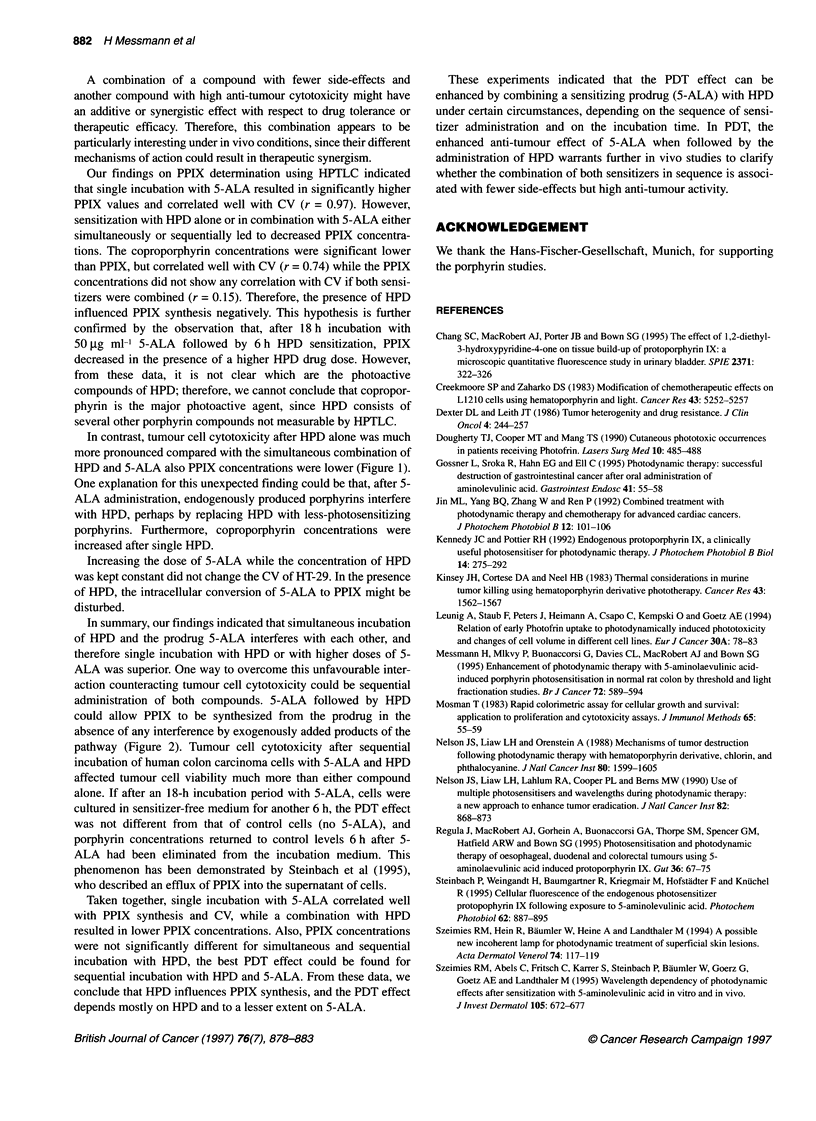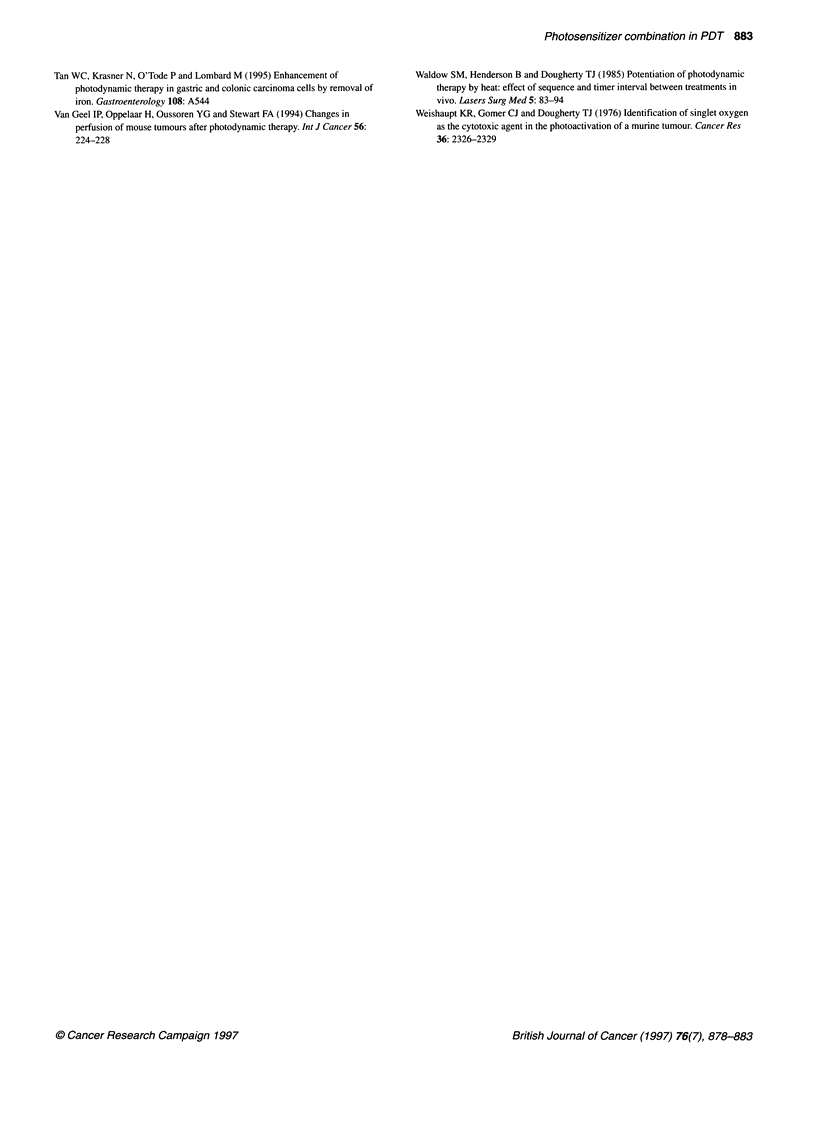# Influence of a haematoporphyrin derivative on the protoporphyrin IX synthesis and photodynamic effect after 5-aminolaevulinic acid sensitization in human colon carcinoma cells.

**DOI:** 10.1038/bjc.1997.478

**Published:** 1997

**Authors:** H. Messmann, M. Geisler, U. Gross, C. Abels, R. M. Szeimies, P. Steinbach, R. KnÃ¼chel, M. Doss, J. SchÃ¶lmerich, A. Holstege

**Affiliations:** Department of Internal Medicine I, University of Regensburg, Germany.

## Abstract

Haematoporphyrin derivatives (HPDs) are potent sensitizers in photodynamic therapy (PDT), associated with prolonged skin photosensitivity. 5-Aminolaevulinic acid (5-ALA), a natural precusor of haem, is converted intracellularly into the photosensitive agent protoporphyrin IX (PPIX), causing direct cytotoxicity after laser light irradiation but limited skin photosensitivity over 1-2 days and higher tumour selectivity. Unfortunately, the use of 5-ALA in PDT has been shown to cause only superficial tissue necrosis. Therefore, a combination of HPD and 5-ALA could be of great clinical value in the treatment of tumours if a synergistic effect of both sensitizers on tumour cell necrosis with less skin photosensitivity could be demonstrated. Human colon adenocarcinoma cells (HT-29) were cultured with either HPD or 5-ALA alone, simultaneously for 24 h with 5-ALA and HPD or in succession with 5-ALA (18 h) followed by HPD (6 h at different concentrations. Intracellular PPIX concentrations were determined by high-performance thin-layer chromatography. Furthermore, PDT was performed with an incoherent light source (lambda = 580-740 nm) using a light dose of 30 J cm(-2) and an output power of 40 mW cm(-2). The intracellular PPIX concentration correlated well with 5-ALA drug dose and incubation time and was highest after single 5-ALA sensitization. In the presence of HPD, either simultaneously or sequentially, PPIX decreased significantly. The PDT effect after simultaneous incubation with both sensitizers for 24 h was not superior to incubation with HPD alone. If 5-ALA incubation (18 h) was followed by HPD (6 h) cytotoxicity after PDT was higher than with either single drug. 5-ALA (80 microg ml(-1)) led to a decrease in tumour cell viability by 40%. A similar effect could be observed when 5-ALA and HPD were sequentially combined allowing for a reduction of the 5-ALA dose from 80 microg ml(-1) in the absence of HPD to 60 microg ml(-1) and 5 microg ml(-1) together with 0.5 microg ml(-1) and 2 microg ml(-1) HPD respectively. We speculate that the enhanced PDT effect after the combined administration of 5-ALA and HPD to cultures of colon carcinoma cells should be even more impressive in the tumour in vivo, since HPD primarily targets the tumour microvasculature and secondarily tumour cells.


British Journal of Cancer (1997) 76(7), 878–883
© 1997 Cancer Research Campaign

### Influence of a haematoporphyrin derivative on the protoporphyrin IX synthesis and photodynamic effect after 5-aminolaevulinic acid sensitization in human colon carcinoma cells

**H Messmann[1], M Geisler[1], U Groß[2], C Abels[3], R-M Szeimies[3], P Steinbach[4], R Knüchel[4], M Doss[2], J Schölmerich[1] and A Holstege[1]**

Department of [1]Internal Medicine I, University of Regensburg; [2]Division of Clinical Biochemistry, Marburg; Departments of [3]Dermatology and [4]Pathology, University of Regensburg, Regensburg, Germany

**Summary** Haematoporphyrin derivatives (HPDs) are potent sensitizers in photodynamic therapy (PDT), associated with prolonged skin photosensitivity. 5-Aminolaevulinic acid (5-ALA), a natural precusor of haem, is converted intracellularly into the photosensitive agent protoporphyrin IX (PPIX), causing direct cytotoxicity after laser light irradiation but limited skin photosensitivity over 1–2 days and higher tumour selectivity. Unfortunately, the use of 5-ALA in PDT has been shown to cause only superficial tissue necrosis. Therefore, a combination of HPD and 5-ALA could be of great clinical value in the treatment of tumours if a synergistic effect of both sensitizers on tumour cell necrosis with less skin photosensitivity could be demonstrated. Human colon adenocarcinoma cells (HT-29) were cultured with either HPD or 5-ALA alone, simultaneously for 24 h with 5-ALA and HPD or in succession with 5-ALA (18 h) followed by HPD (6 h at different concentrations. Intracellular PPIX concentrations were determined by high-performance thin-layer chromatography. Furthermore, PDT was performed with an incoherent light source ($\lambda$ = 580–740 nm) using a light dose of 30 J $cm^{-2}$ and an output power of 40 mW $cm^{-2}$. The intracellular PPIX concentration correlated well with 5-ALA drug dose and incubation time and was highest after single 5-ALA sensitization. In the presence of HPD, either simultaneously or sequentially, PPIX decreased significantly. The PDT effect after simultaneous incubation with both sensitizers for 24 h was not superior to incubation with HPD alone. If 5-ALA incubation (18 h) was followed by HPD (6 h) cytotoxicity after PDT was higher than with either single drug. 5-ALA (80 $\mu$g $ml^{-1}$) led to a decrease in tumour cell viability by 40%. A similar effect could be observed when 5-ALA and HPD were sequentially combined allowing for a reduction of the 5-ALA dose from 80 $\mu$g $ml^{-1}$ in the absence of HPD to 60 $\mu$g $ml^{-1}$ and 5 $\mu$g $ml^{-1}$ together with 0.5 $\mu$g $ml^{-1}$ and 2 $\mu$g $ml^{-1}$ HPD respectively. We speculate that the enhanced PDT effect after the combined administration of 5-ALA and HPD to cultures of colon carcinoma cells should be even more impressive in the tumour in vivo, since HPD primarily targets the tumour microvasculature and secondarily tumour cells.

**Keywords:** photodynamic therapy; sensitizer combination; 5-aminolaevulinic acid; haematoporphyrin derivatives

Photodynamic therapy (PDT) is a promising non-thermal technique for inducing selective necrosis in neoplastic tissues with laser light after prior administration of a photosensitizing drug. The drug is activated by light of a specific wavelength matching its absorption spectrum. In the presence of oxygen, the activated sensitizer induces the formation of reactive oxygen species, in particular singlet oxygen (Weishaupt et al, 1976). The ideal photosensitizer accumulates almost exclusively in tumours with minimal retention in the surrounding normal tissue.

The currently clinically approved photosensitizers [haematoporphyrin derivative (HPD) and its purified components] are powerful sensitizers but with low tumour selectivity and prolonged skin photosensitivity (Dougherty et al, 1990). In addition to tumour cell-directed necrosis induced by PDT, HPD additionally accumulates in endothelial cells (Leunig et al, 1994), allowing destruction of the tumour vascular system (van Geel et al, 1994).

*Received 22 April 1996*
*Revised 14 February 1997*
*Accepted 25 March 1997*

*Correspondence to:* H Messmann, Department of Internal Medicine I, University of Regensburg, 93042 Regensburg, Germany

5-Aminolaevulinic acid (5-ALA) is not a sensitizer by itself but represents a naturally occurring precursor for the haem biosynthetic pathway and has been used as a photosensitizing prodrug that is metabolized intracellularly to porphyrins, known to be efficient photosensitizers (Kennedy et al, 1990). 5-ALA has several advantages when compared with other sensitizers in PDT: 5-ALA-induced porphyrins are rapidly eliminated from the body, limiting skin sensitivity to 1–2 days (Regula et al, 1995). In addition, 5-ALA shows a better uptake and metabolism by tumour cells than by their parent cells. The drug is effective in PDT within the gastrointestinal tract (Gossner et al, 1995; Regula et al, 1995). Unfortunately, PDT induces only superficial tumour necrosis in patients sensitized with 5-ALA (Regula et al, 1995). Accordingly, several approaches have been made to improve the PDT effect of 5-ALA. Laser light fractionation enhances the effect of PDT after 5-ALA sensitization (Messmann et al, 1995). Furthermore, the PDT effect after 5-ALA sensitization is wavelength dependent, and best results can be achieved by irradiating at 635 nm (Szeimies et al, 1995). The combination of 5-ALA with desferrioxamine, an enzyme inhibitor of ferrochelatase, increased the concentration of PPIX in normal mucosa of the urinary bladder (Chang et al, 1994). This approach also led to a better PDT effect upon gastric and colonic carcinoma cells (Tan et al, 1994).

The combination of two differentially acting sensitizers, such as 5-ALA and HPD, could be another promising approach to enhance PDT efficacy. Owing to its mechanism, 5-ALA is intracellularly converted into PPIX and therefore induces direct phototoxicity after PDT. In contrast, HPD is mainly targeted at tumour vessels by accumulating in vascular endothelial cells enabling indirect tumour destruction (Leunig et al, 1994). We wanted to clarify the influence of a haematoporphyrin derivative on the PP IX synthesis after 5-ALA sensitization and whether the PDT effect after 5-ALA sensitization can be enhanced by its combination with low-dose HPD in vitro experiments using cultured human colonic adenocarcinoma cells.

If successful, additional administration of low-dose HPD (e.g. 0.5 mg $kg^{-1}$ i.v.) after 5-ALA sensitization could result in better clinical PDT effects than with single 5-ALA application, without considerable prolongation of skin photosensitivity.

### MATERIAL AND METHODS

### Cells, medium and chemicals

Undifferentiated colon adenocarcinoma cells (HT-29) were maintained as a monolayer culture in Dulbecco's modified Eagle medium (DMEM; Biochrom, Berlin, Germany), supplemented with 10% fetal calf serum (FCS; Gibco, Eggenstein, Germany), 1% sodium pyruvate, 1% non-essential amino acids and penicillin/streptomycin (100 IU $ml^{-1}$/100 mg $ml^{-1}$) (all Gibco) at 37°C in a 5% carbon dioxide atmosphere. 5-ALA (Merck, Darmstadt, Germany) and Photosan-3 (haematoporphyrin derivative; Seelab, Wesselburenkoog, Germany) were dissolved in phosphate-buffered saline (Biochrom) at a concentration of 1 mg $ml^{-1}$. Aliquots were diluted in DMEM to produce in vitro concentrations ranging from 0.1 to 100 μg $ml^{-1}$, and these were used within 24 h.

### Incubation of cells

When growing exponentially, cells were harvested by enzymatic disaggregation with 0.5% trypsin-EDTA, washed with phosphate-buffered saline (PBS), resuspended in DMEM and seeded out in Falcon tubes. After 1 week, cells were incubated with 5-ALA (5 and 50 μg $ml^{-1}$) for 18 or 24 h with or without HPD (0.5 and 5 μg $ml^{-1}$) simultaneously (24 h) or sequentially (6 h). After sensitization, cells were washed in PBS, trypsinated and centrifuged at 1450 r.p.m. These cells were used for the determination of intracellular PPIX concentration using high-performance thin-layer chromatography (HPTLC). In another set of experiments, cells were seeded out at a concentration of 15 000 cells per well in 96-well dishes (Fa. Greiner, Frickenhausen, Germany) for PDT. After a 24-h preincubation period, cells were first incubated simultaneously with 5-ALA (0, 1, 5, 10, 40, 60, 80 and 100 μg $ml^{-1}$) and HPD (0, 0.5, 2 and 5 μg $ml^{-1}$) for 24 h. Furthermore, cells were incubated for 18 h with 5-ALA, washed in PBS, followed by another incubation period of 6 h in sensitizer-free or HPD-containing medium.

### Spectrophotometric quantification of porphyrins by thin-layer chromatography

*Homogenization and esterification*

The pelleted cell material was diluted with 1 ml of PBS, frozen and thawed three times and sonicated for 20 s three times. This extract was frozen in 20-ml tubes and freeze dried. The freeze-dried material was esterified with methanol–sulphuric acid (95:5, v/v) overnight.

*Extraction of the porphyrin methyl esters*

The esterification mixture was extracted with chloroform after the addition of about 5 ml of distilled water. The extraction of the aqueous phase with chloroform was continued until the supernatant no longer fluoresced under UV light (366 nm). The supernatants were combined in a second extraction vessel, shaken and neutralized with an aqueous sodium bicarbonate solution (50 g $l^{-1}$). The upper aqueous phase was withdrawn, and the chloroform extracts were washed twice with about 4 ml of distilled water. The aqueous phase must be neutral after the second washing. The chloroform extract was dried with 2–3 spatula tips of dry sodium sulphate and filtered through a folded paper filter into a flask. The flask was attached to a rotary evaporator, and the chloroform was removed under vacuum at a temperature of 25–30°C.

*High-performance thin-layer chromatography*

The dried porphyrin methyl esters were dissolved in a small volume of chloroform (0.1–0.5 ml) and applied, in stripes, to the silica gel with glass capillaries. The evaporation of the solvent was

**Table 1** Intracellular porphyrin concentrations after sensitization with 5-ALA and/or HPD and corresponding CV

| Sensitizer | Uroporphyrin (pmol $ml^{-1}$ cells) | Coproporphyrin (pmol $ml^{-1}$ cells) | PPIX (pmol $ml^{-1}$ cells) | Total porphyrins (pmol $ml^{-1}$ cells) | CV (%) |
|---|---|---|---|---|---|
| Control (no sensitizer) | 0 | 0 | 0 | 0 | 100 |
| 5-ALA (24 h; 5 μg $ml^{-1}$) | 0 | 0 | 85 | 85 | 93 |
| 5-ALA (24 h; 50 μg $ml^{-1}$) | 0 | 32 | 1313 | 1353 | 65 |
| HPD (24 h; 5 μg $ml^{-1}$) | 0 | 89 | 72 | 161 | 11 |
| 5-ALA (24 h; 5 μg $ml^{-1}$) + HPD (24 h; 5 μg $ml^{-1}$) | 0 | 68 | 85 | 153 | 36 |
| 5-ALA (24 h; 50 μg $ml^{-1}$) + HPD (24 h; 5 μg $ml^{-1}$) | 0 | 59 | 220 | 279 | 34 |
| 5-ALA (18 h; 50 μg $ml^{-1}$) | 23 | 65 | 1030 | 1118 | 78 |
| 5-ALA (18 h; 50 μg $ml^{-1}$) + medium (6 h) | 23 | 32 | 476 | 531 | 88 |
| HPD (6 h; 0.5 μg $ml^{-1}$) | 0 | 32 | 40 | 72 | 90 |
| HPD (6 h; 5 μg $ml^{-1}$) | 0 | 63 | 36 | 99 | 21 |
| 5-ALA (18 h; 50 μg $ml^{-1}$) + HPD (6 h; 0.5 μg $ml^{-1}$) | 0 | 0 | 553 | 553 | 63 |
| 5-ALA (18 h; 50 μg $ml^{-1}$) + HPD (6 h; 5 μg $ml^{-1}$) | 0 | 64 | 178 | 242 | 18 |

© Cancer Research Campaign 1997

accelerated by a stream of air from a cold-air hair dryer placed about 10 cm above the sheet.

The thin-layer sheets were placed in a chromatography chamber and a short prerun with chloroform methanol (120:20, v/v), 1 cm, was performed. Then the chromatogram must be dried completely for 40 min at room temperature in the dark, or 12 min under a cold-air hair dryer. Chromatography in petroleum ether (40–60°C)–diethyl ether (3:1, v/v) follows to the upper edge of the sheet. Afterwards, the thin-layer sheets were run in benzene–ethyl acetate–methanol (85:13.5:1.5 by volume), 8–14 cm.

### *Spectrophotometric analysis*

In preparation for spectrophotometric analysis, the porphyrin methyl esters were eluted from the thin-layer sheets. First, the red-fluorescing zones were circled with a scalpel, scraped off the plate, transferred into a flask, eluted with a defined volume of chloroform and analysed spectrophotometrically between 650 and 350 nm. Calculation of the concentrations was done using the millimolar extinction coefficient.

### Irradiation procedure of HT-29 cells

At the end of the incubation period, the medium containing the sensitizer was removed and replaced by PBS. The cells were immediately photoirradiated using a PDT 1200 lamp (Waldmann Medizintechnik, VS-Schwenningen, Germany) emitting incoherent light. The light source was a 1200-W metal halogen lamp (MSR 1200; Philips, Eindhoven, The Netherlands); emission of 580–740 nm radiation was achieved by using dichroic cut-off filters (DT Rot and Calflex-3000; Balzers Optik, Nuremberg, Germany). Irradiation was performed using an output power of 40 mW $cm^{-2}$. After photoirradiation, PBS was replaced with culture medium and the dishes were incubated at 37°C for another 48 h.

**Figure 1** PDT of HT-29 colon carcinoma cells after simultaneous incubation with 5-ALA and HPD at different drug doses for 24 h. Irradiation was performed with 30 J $cm^{-2}$ and an output power of 40 mW $cm^{-2}$. Each point represents the mean ± s.e. of three experiments. Cell viability was determined as described in Material and methods

### Assessment of cell viability

Mitochondrial activity as a parameter for cell viability was determined using the MTT (3-[(4,5-dimethylthiazol-2-yl)-2,5-diphenyltetrazolium bromide]) assay (Mosman et al, 1983). After the addition of 100 µl of a 0.05% MTT solution (Sigma, Deisenhofen, Germany) to each well, the dishes were incubated for 4 h at 37°C. Aliquots (100 µl) of 20% sodium dodecyl sulphate (SDS) were added to each well in order to dissolve the MTT crystals. After a 20-h incubation period at 37°C, optical density was measured on an enzyme-linked immunosorbent assay (ELISA) plate reader (Molecular Devices, USA) at 540 nm to assess cell viability. Optical density as a measure of cell viability (CV) was compared with that of the unirradiated control group without sensitizer application, which was set to 100%.

### Statistical analysis

Each individual experiment was repeated at least three times. Data are presented as the means of at least nine measurements with standard errors. Differences were tested for statistical significance with the one-way repeated measures ANOVA or the one-way ANOVA on ranks. Statistically significant differences were assumed at $P < 0.05$.

### RESULTS

### Intracellular porphyrin concentrations after sensitization with 5-ALA and HPD

The intracellular porphyrin concentrations of uroporphyrin, coproporphyrin and PPIX, as well as the total porphyrin concentrations, are shown in Table 1. Highest PPIX concentrations were achieved after single incubation with 5-ALA, depending on incubation time and 5-ALA dose. Single incubation with HPD was followed by lower PPIX concentrations compared with 5-ALA sensitization, but relatively high values of coproporphyrins.

PPIX concentrations were identical after simultaneous incubation with 5-ALA and HPD (5 µg $ml^{-1}$ each) for 24 h or single 5-ALA (5 µg $ml^{-1}$) incubation, which resulted in 85 pmol $ml^{-1}$ cells, but were significantly higher with 50 µg $ml^{-1}$ 5-ALA and 5 µg $ml^{-1}$ HPD (220 pmol $ml^{-1}$ cells; $P < 0.01$). However, while 24 h incubation with 50 µg $ml^{-1}$ 5-ALA yielded 1313 pmol $ml^{-1}$ cells of PPIX, 5 µg $ml^{-1}$ HPD induced only 72 pmol $ml^{-1}$ cells of PPIX. Thus, the combined incubation resulted not in an additional effect of the PPIX concentration but in a significantly reduced PPIX value of 220 pmol $ml^{-1}$ cells.

A sequential incubation of 5-ALA and HPD also showed lower PPIX concentrations compared with single 5-ALA sensitization. However, the higher the HPD concentration, the lower the PPIX concentrations.

### PDT effect on HT-29 cells after sensitization with either 5-ALA or HPD alone

Incubation of HT-29 cells with 0.5 µg $ml^{-1}$ HPD alone for 24 h yielded a CV of 70%, whereas 5 µg $ml^{-1}$ HPD yielded a CV of only 11% ($P < 0.001$) (Figure 1). PDT of HT-29 cells after 5-ALA sensitization alone resulted in an almost linear decrease of CV with increasing 5-ALA doses (Figure 1). Comparing the dose of both sensitizers, HPD was more effective than 5-ALA, since

© Cancer Research Campaign 1997

**Figure 2** PDT of HT-29 colon carcinoma cells after sequential incubation with 5-ALA for 18 h and HPD for 6 h. Irradiation was performed with 30 J cm$^{-2}$ and an output power of 40 mW cm$^{-2}$. The vertical lines to the *x*-axis indicate different 5-ALA concentrations in combination with HPD to reduce CV by 40%. Each point represents the mean ± s.e. of three experiments. Cell viability was determined as described in Material and methods

2 µg ml$^{-1}$ HPD induced the same cytotoxic effect of 60% CV as 50 µg ml$^{-1}$ 5-ALA (Figure 1).

### PDT effect on HT-29 cells after simultaneous sensitization with 5-ALA and HPD

Simultaneous incubation of HPD and 5-ALA turned out to be less cytotoxic than exclusive sensitization with HPD. HPD alone (5 µg ml$^{-1}$) reduced CV to 11% after irradiation, while its combination with 1 µg ml$^{-1}$ 5-ALA decreased cytotoxicity by 20%. Similar effects could be demonstrated for each of the HPD concentrations combined with 5-ALA (Figure 1).

Moreover, increasing the concentration of 5-ALA without changing the dose of HPD was not followed by a better PDT effect. The lowest concentration of 5-ALA (1 µg ml$^{-1}$) used in combination with 5 µg ml$^{-1}$ HPD had the same cell-killing efficacy as 50 µg ml$^{-1}$ 5-ALA together with 5 µg ml$^{-1}$ HPD, resulting in a CV of 35% or 34% respectively. Accordingly, the CV after simultaneous incubation with 5-ALA and HPD appeared to be independent of 5-ALA concentrations, but was mostly determined by HPD. For example, the combination of 50 µg ml$^{-1}$ 5-ALA with 5 µg ml$^{-1}$ HPD induced a more than 40% higher cell cytotoxicity compared with a tenfold lower dose of HPD in this combination with 5-ALA (Figure 1).

Comparing the PDT effect on cell viability after sensitization with low doses of 5-ALA (1–40 µg ml$^{-1}$) alone or in combination with HPD, the combined administration of both sensitizers seems to be superior to the isolated use of 5-ALA. This effect was independent of the 5-ALA concentration and therefore was only influenced by the more powerful sensitizer HPD.

In contrast, higher doses of 5-ALA alone (≥ 80 µg ml$^{-1}$) were more toxic than their combination with 0.5 or 2 µg ml$^{-1}$ HPD. Accordingly, PDT with 100 µg ml$^{-1}$ 5-ALA reduced the CV to 3%, whereas its combination with 0.5 µg ml$^{-1}$ HPD reduced cytotoxicity by almost 71% (Figure 1).

### PDT effect on HT-29 cell viability after successive exposure to 5-ALA and HPD

Simultaneous incubation of 5-ALA and HPD indicated that both sensitizers can interfere with each other, thus reducing the PDT effect compared with single incubation with either HPD or 5-ALA (> 80 µg ml$^{-1}$). Therefore, the incubation period of 24 h was split into 18 h with 5-ALA followed by 6 h with HPD to ensure that cells were photoradiated after the same total incubation time.

Incubation with 5-ALA (18 h) followed by a 6-h period in sensitizer-free medium showed no difference in CV when compared with unsensitized control cells (no 5-ALA) (Figure 2). However, 5-ALA (18 h) followed by HPD (6 h) reduced the CV after irradiation compared with either drug alone. Single incubation with 2 µg ml$^{-1}$ HPD reduced the CV to 60%, and exclusive sensitization with 100 µg ml$^{-1}$ 5-ALA resulted in a CV of 47%, whereas sequential administration of both compounds yielded a 29% CV of HT-29 cells.

The 5-ALA dose ($LD_{40}$) needed to induce a lethality of 40% can be lowered to 57 µg ml$^{-1}$ and 5 µg ml$^{-1}$ 5-ALA, when sequentially combined with 0.5 and 2 µg ml$^{-1}$ of HPD respectively (Figure 2).

### Correlation of intracellular porphyrins and CV

After single incubation with 5-ALA, the CV correlated well with PPIX concentration ($r$ = 0.97). However, in combination with HPD, the co-relationship for PPIX and CV decreased to 0.15, while the coproporphyrin concentration correlated better ($r$ = 0.74).

### DISCUSSION

The clinical and pharmacological use of combination chemotherapy has been widely studied in animal models (Dexter et al, 1986). The combination of antineoplastic drugs has several advantages over single drug treatment: (1) increasing therapeutic synergism by exploiting different mechanisms of action; (2) increasing patient tolerance by minimizing side-effects of drugs owing to lower doses of each individual compound; and (3) preventing or delaying the emergence of resistant cell clones.

In vitro and in vivo studies have indicated enhanced tumour cytotoxicity when PDT was combined with hyperthermia (Kinsey et al, 1983; Waldow et al, 1985) or chemotherapy (Creekmore et al, 1983; Jin et al, 1992). Combination of different sensitizers is another approach to increasing the PDT effect. By combining haematoporphyrins (2.5 mg kg$^{-1}$) with meso-tetra-(4-sulphonato-phenyl)-porphine (TPPS4; 2.5 mg kg$^{-1}$) and irradiation at the appropriate wavelength for each sensitizer, enhanced tumour eradication compared with either sensitizer alone (5 mg kg$^{-1}$) was achieved (Nelson et al, 1990).

5-ALA sensitization seems to represent an attractive approach to improve PDT by reducing skin photosensitivity and increasing tumour selectivity (Regula et al, 1995). However, its use in cancer treatment is limited by only superficial tumour necrosis (Regula et al, 1995). This problem prompted us to evaluate the PDT effect of a combination of 5-ALA with a second photosensitizer, HPD.

5-ALA is converted intracellularly to PPIX and other porphyrins, which represent the actual phototoxic agents inducing direct tumour cell necrosis. PDT after HPD pretreatment destroys the tumour both directly, and mainly indirectly, by targeting the microvasculature of neoplastic tissue (Nelson et al, 1988; Leunig et al, 1994).

*© Cancer Research Campaign 1997*

A combination of a compound with fewer side-effects and another compound with high anti-tumour cytotoxicity might have an additive or synergistic effect with respect to drug tolerance or therapeutic efficacy. Therefore, this combination appears to be particularly interesting under in vivo conditions, since their different mechanisms of action could result in therapeutic synergism.

Our findings on PPIX determination using HPTLC indicated that single incubation with 5-ALA resulted in significantly higher PPIX values and correlated well with CV ($r$ = 0.97). However, sensitization with HPD alone or in combination with 5-ALA either simultaneously or sequentially led to decreased PPIX concentrations. The coproporphyrin concentrations were significant lower than PPIX, but correlated well with CV ($r$ = 0.74) while the PPIX concentrations did not show any correlation with CV if both sensitizers were combined ($r$ = 0.15). Therefore, the presence of HPD influenced PPIX synthesis negatively. This hypothesis is further confirmed by the observation that, after 18 h incubation with 50 $\mu$g $ml^{-1}$ 5-ALA followed by 6 h HPD sensitization, PPIX decreased in the presence of a higher HPD drug dose. However, from these data, it is not clear which are the photoactive compounds of HPD; therefore, we cannot conclude that coproporphyrin is the major photoactive agent, since HPD consists of several other porphyrin compounds not measurable by HPTLC.

In contrast, tumour cell cytotoxicity after HPD alone was much more pronounced compared with the simultaneous combination of HPD and 5-ALA also PPIX concentrations were lower (Figure 1). One explanation for this unexpected finding could be that, after 5-ALA administration, endogenously produced porphyrins interfere with HPD, perhaps by replacing HPD with less-photosensitizing porphyrins. Furthermore, coproporphyrin concentrations were increased after single HPD.

Increasing the dose of 5-ALA while the concentration of HPD was kept constant did not change the CV of HT-29. In the presence of HPD, the intracellular conversion of 5-ALA to PPIX might be disturbed.

In summary, our findings indicated that simultaneous incubation of HPD and the prodrug 5-ALA interferes with each other, and therefore single incubation with HPD or with higher doses of 5-ALA was superior. One way to overcome this unfavourable interaction counteracting tumour cell cytotoxicity could be sequential administration of both compounds. 5-ALA followed by HPD could allow PPIX to be synthesized from the prodrug in the absence of any interference by exogenously added products of the pathway (Figure 2). Tumour cell cytotoxicity after sequential incubation of human colon carcinoma cells with 5-ALA and HPD affected tumour cell viability much more than either compound alone. If after an 18-h incubation period with 5-ALA, cells were cultured in sensitizer-free medium for another 6 h, the PDT effect was not different from that of control cells (no 5-ALA), and porphyrin concentrations returned to control levels 6 h after 5-ALA had been eliminated from the incubation medium. This phenomenon has been demonstrated by Steinbach et al (1995), who described an efflux of PPIX into the supernatant of cells.

Taken together, single incubation with 5-ALA correlated well with PPIX synthesis and CV, while a combination with HPD resulted in lower PPIX concentrations. Also, PPIX concentrations were not significantly different for simultaneous and sequential incubation with HPD, the best PDT effect could be found for sequential incubation with HPD and 5-ALA. From these data, we conclude that HPD influences PPIX synthesis, and the PDT effect depends mostly on HPD and to a lesser extent on 5-ALA.

These experiments indicated that the PDT effect can be enhanced by combining a sensitizing prodrug (5-ALA) with HPD under certain circumstances, depending on the sequence of sensitizer administration and on the incubation time. In PDT, the enhanced anti-tumour effect of 5-ALA when followed by the administration of HPD warrants further in vivo studies to clarify whether the combination of both sensitizers in sequence is associated with fewer side-effects but high anti-tumour activity.

### ACKNOWLEDGEMENT

We thank the Hans-Fischer-Gesellschaft, Munich, for supporting the porphyrin studies.

### REFERENCES

Chang SC, MacRobert AJ, Porter JB and Bown SG (1995) The effect of 1,2-diethyl-3-hydroxypyridine-4-one on tissue build-up of protoporphyrin IX: a microscopic quantitative fluorescence study in urinary bladder. *SPIE* **2371**: 322–326

Creekmoore SP and Zaharko DS (1983) Modification of chemotherapeutic effects on L1210 cells using hematoporphyrin and light. *Cancer Res* **43**: 5252–5257

Dexter DL and Leith JT (1986) Tumor heterogenity and drug resistance. *J Clin Oncol* **4**: 244–257

Dougherty TJ, Cooper MT and Mang TS (1990) Cutaneous phototoxic occurrences in patients receiving Photofrin. *Lasers Surg Med* **10**: 485–488

Gossner L, Sroka R, Hahn EG and Ell C (1995) Photodynamic therapy: successful destruction of gastrointestinal cancer after oral administration of aminolevulinic acid. *Gastrointest Endosc* **41**: 55–58

Jin ML, Yang BQ, Zhang W and Ren P (1992) Combined treatment with photodynamic therapy and chemotherapy for advanced cardiac cancers. *J Photochem Photobiol B* **12**: 101–106

Kennedy JC and Pottier RH (1992) Endogenous protoporphyrin IX, a clinically useful photosensitiser for photodynamic therapy. *J Photochem Photobiol B Biol* **14**: 275–292

Kinsey JH, Cortese DA and Neel HB (1983) Thermal considerations in murine tumor killing using hematoporphyrin derivative phototherapy. *Cancer Res* **43**: 1562–1567

Leunig A, Staub F, Peters J, Heimann A, Csapo C, Kempski O and Goetz AE (1994) Relation of early Photofrin uptake to photodynamically induced phototoxicity and changes of cell volume in different cell lines. *Eur J Cancer* **30A**: 78–83

Messmann H, Mlkvy P, Buonaccorsi G, Davies CL, MacRobert AJ and Bown SG (1995) Enhancement of photodynamic therapy with 5-aminolaevulinic acid-induced porphyrin photosensitisation in normal rat colon by threshold and light fractionation studies. *Br J Cancer* **72**: 589–594

Mosman T (1983) Rapid colorimetric assay for cellular growth and survival: application to proliferation and cytotoxicity assays. *J Immunol Methods* **65**: 55–59

Nelson JS, Liaw LH and Orenstein A (1988) Mechanisms of tumor destruction following photodynamic therapy with hematoporphyrin derivative, chlorin, and phthalocyanine. *J Natl Cancer Inst* **80**: 1599–1605

Nelson JS, Liaw LH, Lahlum RA, Cooper PL and Berns MW (1990) Use of multiple photosensitisers and wavelengths during photodynamic therapy: a new approach to enhance tumor eradication. *J Natl Cancer Inst* **82**: 868–873

Regula J, MacRobert AJ, Gorhein A, Buonaccorsi GA, Thorpe SM, Spencer GM, Hatfield ARW and Bown SG (1995) Photosensitisation and photodynamic therapy of oesophageal, duodenal and colorectal tumours using 5-aminolaevulinic acid induced protoporphyrin IX. *Gut* **36**: 67–75

Steinbach P, Weingandt H, Baumgartner R, Kriegmair M, Hofstädter F and Knüchel R (1995) Cellular fluorescence of the endogenous photosensitizer protopophyrin IX following exposure to 5-aminolevulinic acid. *Photochem Photobiol* **62**: 887–895

Szeimies RM, Hein R, Bäumler W, Heine A and Landthaler M (1994) A possible new incoherent lamp for photodynamic treatment of superficial skin lesions. *Acta Dermatol Venerol* **74**: 117–119

Szeimies RM, Abels C, Fritsch C, Karrer S, Steinbach P, Bäumler W, Goerz G, Goetz AE and Landthaler M (1995) Wavelength dependency of photodynamic effects after sensitization with 5-aminolevulinic acid in vitro and in vivo. *J Invest Dermatol* **105**: 672–677

© Cancer Research Campaign 1997

Tan WC, Krasner N, O'Tode P and Lombard M (1995) Enhancement of photodynamic therapy in gastric and colonic carcinoma cells by removal of iron. *Gastroenterology* **108**: A544

Van Geel IP, Oppelaar H, Oussoren YG and Stewart FA (1994) Changes in perfusion of mouse tumours after photodynamic therapy. *Int J Cancer* **56**: 224–228

Waldow SM, Henderson B and Dougherty TJ (1985) Potentiation of photodynamic therapy by heat: effect of sequence and timer interval between treatments in vivo. *Lasers Surg Med* **5**: 83–94

Weishaupt KR, Gomer CJ and Dougherty TJ (1976) Identification of singlet oxygen as the cytotoxic agent in the photoactivation of a murine tumour. *Cancer Res* **36**: 2326–2329

© Cancer Research Campaign 1997